# Machine Learning in Predicting Printable Biomaterial Formulations for Direct Ink Writing

**DOI:** 10.34133/research.0197

**Published:** 2023-07-18

**Authors:** Hongyi Chen, Yuanchang Liu, Stavroula Balabani, Ryuji Hirayama, Jie Huang

**Affiliations:** ^1^ Department of Mechanical Engineering, University College London, London, UK.; ^2^ Department of Computer Science, University College London, London, UK.; ^3^ Wellcome-EPSRC Centre for Interventional Surgical Sciences (WEISS), University College London, London, UK.

## Abstract

Three-dimensional (3D) printing is emerging as a transformative technology for biomedical engineering. The 3D printed product can be patient-specific by allowing customizability and direct control of the architecture. The trial-and-error approach currently used for developing the composition of printable inks is time- and resource-consuming due to the increasing number of variables requiring expert knowledge. Artificial intelligence has the potential to reshape the ink development process by forming a predictive model for printability from experimental data. In this paper, we constructed machine learning (ML) algorithms including decision tree, random forest (RF), and deep learning (DL) to predict the printability of biomaterials. A total of 210 formulations including 16 different bioactive and smart materials and 4 solvents were 3D printed, and their printability was assessed. All ML methods were able to learn and predict the printability of a variety of inks based on their biomaterial formulations. In particular, the RF algorithm has achieved the highest accuracy (88.1%), precision (90.6%), and F1 score (87.0%), indicating the best overall performance out of the 3 algorithms, while DL has the highest recall (87.3%). Furthermore, the ML algorithms have predicted the printability window of biomaterials to guide the ink development. The printability map generated with DL has finer granularity than other algorithms. ML has proven to be an effective and novel strategy for developing biomaterial formulations with desired 3D printability for biomedical engineering applications.

## Introduction

Using three-dimensional (3D) printing for biomedical applications has gained popularity in recent years [1]. It is capable of fabricating complex implants and allows their architecture to be directly customized to be specific to each patient and clinical condition [2–4]. Among 3D printing techniques, direct ink writing (DIW), which involves directly extruding relatively viscous material through a moving nozzle, has been widely adopted in the biomedical field [5–8]. It operates at room temperature, allowing the extrusion of a broad selection of bioactive and smart materials and cells for tissue engineering [9], drug delivery [10], 4D printing [11], and bioprinting [12].

According to the solvent used, there are 2 types of polymeric inks for DIW, aqueous hydrogels and polymer/organic solvent mixtures. Hydrogel polymer networks swell extensively in water and contain several features resembling those of a natural extracellular matrix (ECM), which are desirable for the adhesion and growth of cells [13,14]. Hydrogels are widely used for soft tissue engineering, allowing not only the diffusion of nutrients and cellular waste products but also the encapsulation of cells to make bioinks. The printing bioinks is then termed bioprinting [2,15–18]. Hydrogels made from natural polymers, such as alginate, collagen, and gelatin, have the advantage of resembling native ECM chemically and structurally [19]. On the other hand, hydrogels made from synthetic polymers including Pluronic F127 (F), polyethylene oxide, and polyvinyl alcohol possess the advantages of reproducibility and tailorability of their chemistry and properties [20]. In particular, Pluronic F127 has been used as a smart hydrogel for 3D bioprinting [21], injectable drug delivery [22], and 4D printing [23], due to its thermo-gelling ability, high biocompatibility, and distinctive shear-thinning property [24]. Polymer/organic solvent systems are composed of polymers, often insoluble in water, dissolved in organic solvents (e.g., acetone and dichloromethane [DCM]). After being printed, the organic solvent evaporates rapidly, leaving behind a solidified polymer-based structure. A Food and Drug Administration-approved polymer, polycaprolactone (PCL), has been widely used for 3D printing bone scaffolds due to its high biocompatibility, toughness, and controlled biodegradation rate [6,25,26]. It has also been used to co-print with hydrogels to provide a mechanical framework [27]. In this study, aqueous hydrogel inks are referred to as hydrogel-based inks, while polymer organic solvent inks are termed polymer-based inks.

Functional bioactive fillers can be added to both hydrogel-based and polymer-based inks to improve the viscosity and printability of the inks or the bioactivity and stiffness of the 3D printed structure [28]. In particular, nanomaterials are considered highly efficient fillers in improving functionality including printability due to their high surface area and small size [29–31]. For instance, Laponite nanoclay (LP) is a widely used bioactive viscosity enhancer for bioinks in bioprinting and hydroxyapatite nanoparticles (nHA) are a well-known bioactive filler for bone tissue engineering [32,33]. Bentonite nanoclay, on the other hand, has been demonstrated to be an effective drug delivery system due to its high surface area-to-volume ratio.

Apart from the compatible biological properties, printability is also a fundamental property of an ink for 3D printing. The architecture of the 3D printed product greatly influences its integration with host tissues and function [34]. Murphy and Atala [2] defined printability as the ability of an ink to be deposited with the desired spatial and temporal control, and Ribeiro et al. [35] defined it as the possibility to be extruded and dispensed with a satisfactory degree of shape fidelity (SF). While there are different opinions in the definition of printability, it is well-received that SF is a key aspect of printability and is evaluated as a means of assessing printability [35–37]. Several methods have been raised to assess the printability of hydrogels qualitatively [38,39] or quantitatively [35,40], but there is no consensus on the quantitative assessment [41]. For polymer-based inks, few studies focus on printability quantification. Therefore, the printability development of inks has been a major target for 3D printing in biomedical applications. Currently, the printability development process includes several steps: material selection from a broad range of choices based on the applications, ink formulation with a range of concentrations, rheological characterization of inks, and printability tests of the formulations [41]. These empirical processes require expert knowledge in each field and are time- and resource-consuming, thus hindering the development of optimal 3D printing inks for biomedical engineering. This is a complex challenge, and in order to address these problems, a new approach is required [42].

Machine learning (ML) is a branch of artificial intelligence that is able to fit predictive models to data or discover patterns within it. It is particularly useful for automating data analysis in a time-efficient and reproducible manner, especially for data that are too large and complex for human analysis [43]. ML methods can be classified into 2 main approaches: supervised learning and unsupervised learning [44]. Supervised learning methods are currently the most widely used [45]. They are trained on labeled data to establish the function that connects input variables (*x*) to output variables (*y*) and then make predictions about unlabeled examples. In the biomedical field, ML has been successfully used for medical image analysis and diagnosis [46–48], gene recognition in a DNA sequence [49], protein structures prediction [50,51], biophysical cue screening [52], and data analysis for organ-on-chips [53,54]. Despite the great potential of ML, the black box nature of ML algorithms still hinders its interpretability and thus its use for interdisciplinary research. Consequently, the use of ML for developing 3D printable biomaterials is underrepresented. Therefore, there is an urgent need to address this limitation and utilize the power of ML in processing printability data and developing 3D printing biomaterials for biomedical applications.

Although ML has demonstrated its capability to transform the analysis of large datasets, only a few studies applied ML to the development of biomaterials for 3D printing. Elbadawi et al. [55] conducted an early and innovative study using ML algorithms to predict the printability of polymer filaments from fused deposition modeling technique for drug delivery. However, in this study, printability is defined as the ability to extrude from the nozzle without printing into a 3D shape, while for biomedical applications, SF of printed products is a fundamental aspect of printability as it influences the integration and function of the products. Nadernezhad and Groll [56] reported a pioneering work using random forest (RF) algorithm for predicting printability with rheological properties and revealed a general understanding of how rheology influences printability. However, it also has some limitations. This study only used one algorithm, RF, and the data only contained one hydrogel material, hyaluronic acid. The performance of ML for evaluating biomaterials with different properties for DIW remains to be ascertained. In addition, various rheological tests are needed to predict printability in the study, which can be an empirical process. Therefore, adopting this interdisciplinary approach of using ML for processing of printability data can accelerate the development of 3D printable biomaterials.

To the best of our knowledge, our study is the first to use ML techniques to predict printable biomaterial ink formulations for 3D structures. A total of 210 biomaterial formulations were 3D printed with DIW technique, and the printability of each formulation was classified (yes/no) based on the SF of the printed structures. The biomaterials cover both natural and synthetic polymers with a range of molecular weights, as well as fillers that can provide a range of functionalities such as bioactivity, sustained drug release, and rheological modification. ML algorithms including decision tree (DT), RF, and deep learning (DL) have predicted the printability using the formulations with a train/test ratio of 7:3 with an accuracy of >80%. Furthermore, the printability window of ink formulations was built to guide the development of printable inks. This study paves the way for unleashing ML algorithms in developing printable biomaterials for biomedical applications.

## Results and Discussion

### Printability assessment

**Fig. 1.**
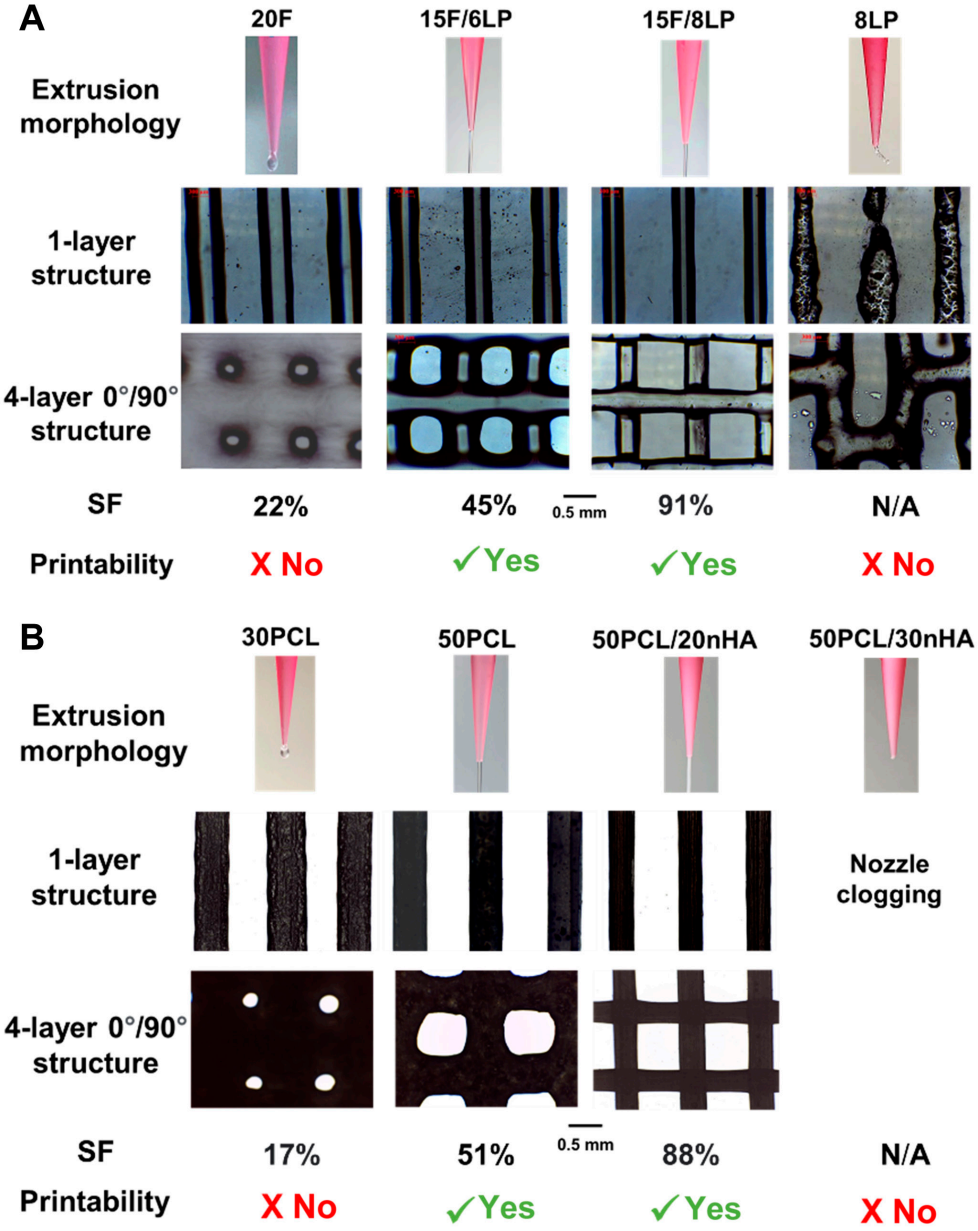
A summary of 3D printing results of (A) hydrogel-based F127/LP inks and (B) polymer-based PCL/nHA inks with DCM as solvent. The number in the ink name refers to wt% for F127/LP inks and w/v% for PCL/nHA inks. The Barus effect (expansion of the inks exiting the nozzle) was observed at low-viscosity inks. The viscosity of inks increases with the concentrations of fillers LP and nHA; at too high viscosity, inks were unextrudable or clogged nozzle, and the printed filaments were prone to fracture. A higher SF of 3D printed filaments and 4-layer 0°/90° scaffold structure was achieved for 15F/8LP and PCL/20nHA inks.

A total of 210 biomaterial formulations were 3D printed into 4-layer 0°/90° scaffold structures, and their printability was defined based on the measured SF of the printed structures (detailed in Materials and Methods). The printability assessment procedure is shown in Fig. 1A and B with examples of F127/LP inks for hydrogel-based inks and PCL/nHA inks for polymer-based inks. During extrusion, inks with low viscosity form wider openings when exiting the nozzle or even droplets instead of filaments (e.g., 20F and 30PCL). This results from the viscoelastic behavior of the inks and the expansion of the inks exiting the nozzle is known as the Barus effect [57]. Additionally, the lower resistance makes the structures more prone to post-printing deformation including the sagging and fusion of the filaments driven by gravity and capillary force respectively. This further makes filaments wider and lower, limiting the scalability of the inks in a layer-by-layer manner. The addition of fillers can enhance the viscosity of inks (e.g., 15F/8LP and PCL/20nHA) and their resistance to deformation, which minimizes the Barus effect to extrude into smooth filaments. The printed structures can better maintain the designated shape and also have higher SF, i.e., up to 91% and 88% for 15F/8LP and PCL/20nHA inks, respectively. This facilitates the building up of the structures in a layer-by-layer manner, thus improving the scalability. If the viscosity of the ink is too high, its brittleness can cause irregular morphology of the deposited filaments (8LP), resulting in fractured filaments or even nozzle clogging (PCL/30nHA).

**Fig. 2.**
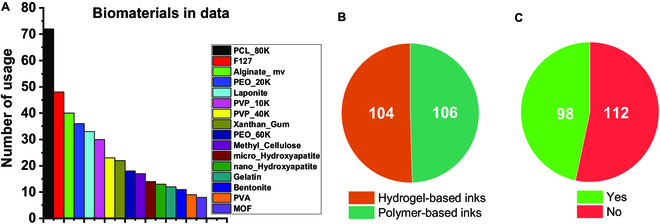
Data summary of the distribution of (A) biomaterials, (B) 2 ink systems, and (C) outputs in terms of the number of usages in the data. The input features include biomaterials and solvents used for the 3D printing process while the output is the printability of the inks.

A variety of biomaterials and solvents were used in this study for ink formulation for 3D printing and printability assessment to generate printability data for training and testing the ML algorithms. In the printability data, the ink formulations are the input, and the printability of the formulations is the output. A summary of the input and output in the printability data is shown in Fig. 2. All biomaterials have appeared 8 times or more in the data and were formulated with a range of concentrations for effectively training the algorithms to recognize the underlying pattern in printability. The ratio between the 2 ink systems and the 2 output results is 104:106 and 98:112, respectively. The data are relatively balanced, which can help prevent bias from the algorithms. The main organic solvent used is DCM. The number of usage for polymeric materials is more than 4 times than that of fillers because a polymer matrix is used in every ink, whereas fillers are used to add functionality (e.g., enhancing viscosity and bioactivity).

### Printability prediction with ML

The printability data were split into a training dataset and a testing dataset with a ratio of 7:3. The algorithms were trained using the training dataset and subsequently tested on the testing dataset unseen to the model to assess their generalization ability [58,59]. The key hyperparameters of the algorithms were tuned to generate the algorithms with varying degrees of complexity and their effect on the prediction performance of the ML models was examined to optimize the algorithms.

**Fig. 3.**
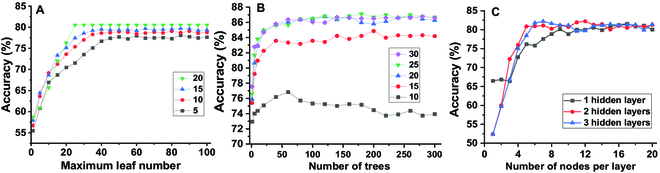
The optimization process of DT, RF, and DL for predicting printability: (A) Effect of the MNL and MNF for splitting (5, 10, 15, and 20) on the performance of the DT algorithm. (B) Effect of both MNL (10, 15, 20, 25, and 30) in DTs and the number of DTs in the RF algorithm on its performance. (C) Effect of the number of nodes in hidden layers and number of hidden layers in the DL algorithm on its performance.

The DT algorithm uses a DT classifier composed of nodes and branches that lead to other nodes and ultimately a leaf (terminal node) where classification is assigned. Part of the DT is shown in Fig. S1. The maximum number of features (MNF) to consider for splitting branches and the maximum number of leaves (MNL) influence the prediction accuracy as shown in Fig. 3A. As the MNL increases, accuracy increases until convergence due to better fitting to the data. As the MNF increases, the convergence was reached with lower MNL and the accuracy at convergence increases. With the MNF set at 20, DT reaches the highest accuracy of 80.5% at an MNL of 20 or above. The accuracy of 80.5% also remains constant with a further increase of MNL above 20 indicating that the DT has been completely developed and ceased expanding further.

The RF algorithm consists of an ensemble of DTs and the MNF for each tree was set as 20. The MNL in each tree and the number of DTs in RF influence its accuracy, as shown in Fig. 3B. The accuracy of RF with a single tree (73% to 77.6%) is lower than that of DT (80.5%) as the training data for a single tree omit, on average, 36.8% of samples in the whole training data from the bootstrap sampling. However, as the number of trees increases, the accuracy of RF increases until convergence is reached due to the inclusion of diverse DTs trained with different bootstrap data. This can enhance the complexity of the predictive model, allowing it to learn more effectively from the complex training data. When the number of trees reaches 5 and the MLN is 20 or above, the accuracy of RF (80.7% to 82.8%) surpasses DT (80.5%). It is noteworthy that when the MNL in each tree is as low as 10, as the number of trees increases past the convergence, the accuracy of the RF model decreases to an extent. This is likely due to overfitting when the complex model fits well to the training dataset but does not generalize well to the testing dataset. When MNL is 15 or above, accuracy does not decrease as the number of trees increases beyond convergence. The reason is that a higher MNL allows for more adequately developed DTs with reduced error in fitting the training dataset. Consequently, the RF model is able to generate large numbers of DTs with high diversity using the bootstrap sampling method while inducing less error to mitigate the issue of overfitting. Due to the random nature of the RF algorithm, the accuracy changes within a boundary with the increase in the number of trees after reaching convergence. The accuracy is highest (88.1%) at 180 DTs with the MNF for splitting in DTs set at 25.

The DL algorithm consists of artificial neural networks (ANNs) with an input layer, hidden layers, and an output layer. Both the number of nodes in hidden layers and the number of hidden layers influence the performance of the algorithm, as shown in Fig. 3C. As the number of nodes per layer increases, the accuracy increases up to a threshold number of nodes: 17 nodes for a 1-layer structure, 12 nodes for a 2-layer structure, and 7 nodes for a 3-layer structure. Below this threshold, the algorithm forms a more complex model with the inclusion of more nodes to learn the training data. Increasing the number of nodes above the threshold decreases accuracy, which is likely due to overfitting. The increase in the number of hidden layers lowers the threshold number of nodes in each layer for convergence as the total node number and complexity of the network structure increase. The accuracy is highest (82.2%) with 3 hidden layers, with 7 nodes in each layer.

**Fig. 4.**
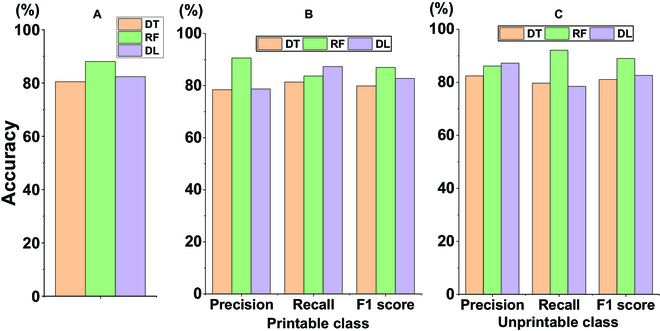
Evaluation metrics of the optimized ML models for predicting printability of biomaterial formulations including (A) prediction accuracy, precision, recall, and F1 score of the printable class (B) and unprintable class (C).

The evaluation metrics including accuracy, precision, recall, and F1 score (as detailed in Materials and Methods) were used to assess the DT, RF, and DL algorithms with the optimized setup as shown in Fig. 4. All 3 algorithms achieved >80% accuracy, with RF having the highest accuracy (88.1%). For predicting the printable class, RF has the highest precision (90.6%), confirming that the predicted printable inks have the highest chance to be useful, which could save researchers’ precious time and resources. On the other hand, although DL has lower precision than RF, it has the highest recall (87.3%), meaning that it has the best chance of predicting printable inks as printable, decreasing the chance of losing printable formulations. This could be very useful for ink development when the pool of printable inks is limited. RF also has the highest F1 score (87.0%), indicating that it had the overall best performance in precision and recall when predicting printable inks. DL has the lowest difference between precision and recall, indicating a balanced performance. There exists a trade-off between precision, which enhances the efficiency by ensuring the printability of inks predicted as printable, and recall, which minimizes the risk of omitting inks that are printable.

The evaluating metrics of the ML algorithms for predicting the unprintable class are shown in Fig. 4C. DL demonstrates the highest precision (87.2%), meaning that the inks predicted as unprintable are the most likely to be unusable. RF has the highest recall (92.1%), indicating that it has the highest coverage for screening out unprintable inks. RF also has the highest F1 score (89.0%), indicating that it had the best overall performance predicting unprintable inks.

RF has shown to have the overall best performance predicting printability, whereas DL has a higher recall when predicting the original dataset, and the highest precision when predicting the inverse dataset. Hence, the choice of algorithms for selecting inks for future ink development should be subject to the evaluation of trade-offs specific to the situation.

### Printability window prediction with ML

**Fig. 5.**
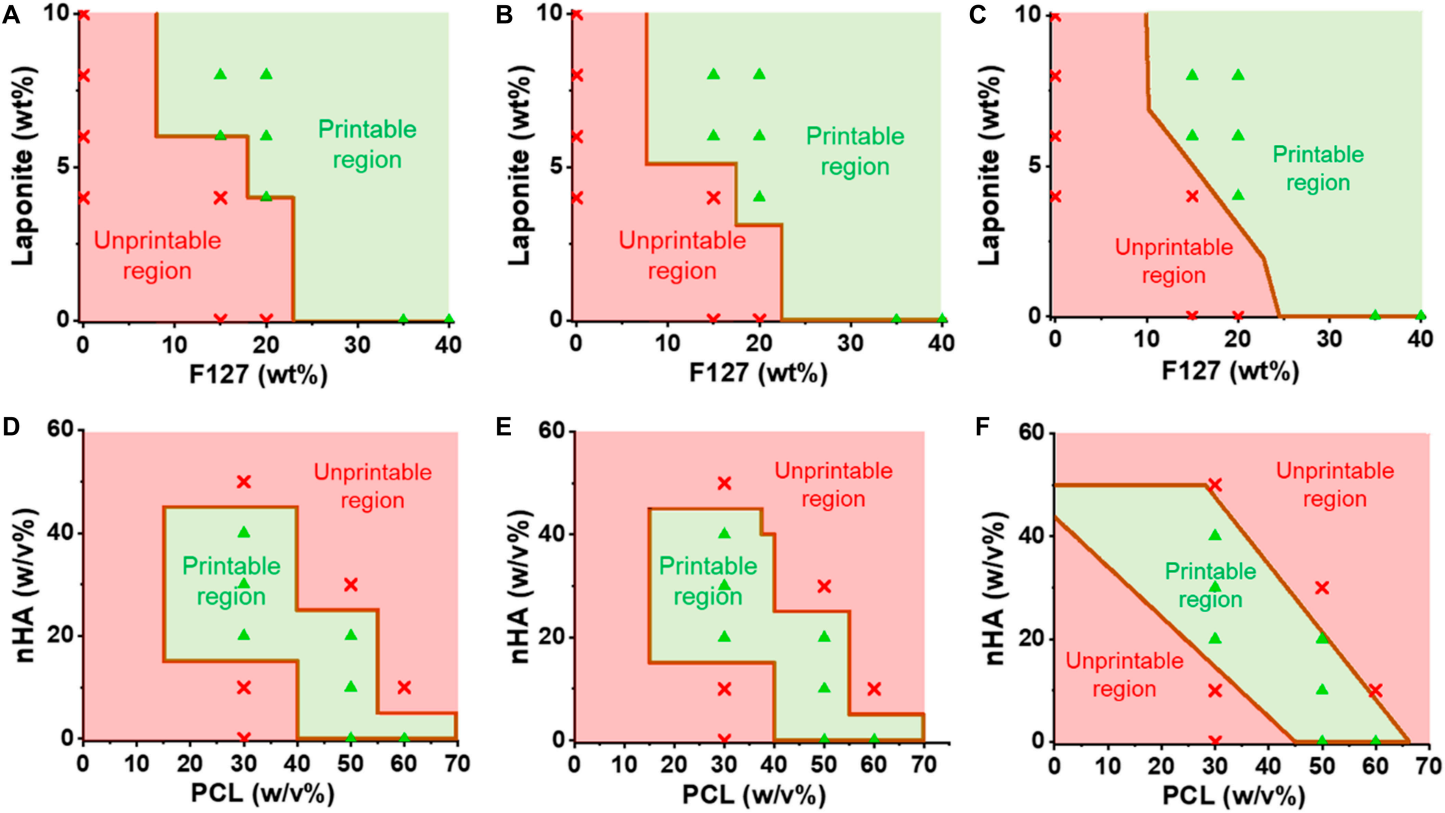
Printability map of both hydrogel-based and polymer-based inks predicted by ML algorithms. (A to C) The printability map of F127/LP hydrogel nanocomposites predicted by DT, RF, and DL, respectively. (D to F) The printability map of PCL/nHA in DCM polymer nanocomposite inks predicted with DT, RF, and DL, respectively. The green triangles and red crosses mark the printable and unprintable formulations, respectively, in the training data. The green and red areas are the predicted printable and unprintable regions, respectively, mapped from the testing results.

After training and tuning, the ML algorithms are capable of predicting the printability window of hydrogel-based and polymer-based inks to guide ink formulations. All 210 formulations were used as training data for the optimized ML algorithms. A total of 45,511 and 350,001 testing formulations were used for F127/LP inks and PCL/nHA inks, respectively. The predicted printability maps of F127/LP hydrogel nanocomposites and PCL/nHA polymer nanocomposites are shown in Fig. 5.

For the predicted printability maps of F127/LP inks, there is a threshold concentration of F127 in terms of a lower limit for formulating printable inks (8.0 wt% for DT, 7.6 wt% for RF, and 9.82 wt% for DL) as shown in Fig. 5A to C. All inks below the threshold value are predicted as unprintable. Above the threshold value, when the concentration of either F127 or Laponite increases, the printable range of the other material tends to increase. This threshold concentration corresponds to the formulation that meets the lowest rheological/printability requirements to be considered as printable in practice. Inks below the threshold F127 concentrations either do not have enough stiffness to retain the structure post-printing (e.g., 20F) or are too brittle from high LP concentrations but lack F127 hydrogel matrix to provide ductility and suffer from filament fractures (e.g., 8LP) as shown in Fig. 1A.

In the printability maps of F127/LP inks predicted by DT and RF (Fig. 5A and B), the boundary regarding LP concentrations consists of 2 concentrations. They are 4.0 wt% and 6.0 wt% for DT and 3.1 wt% and 5.1 wt% for RF, respectively. The possible reason is that there are 2 LP concentrations on the boundary in the training data and they appear in the questions in the trees in DT and RF algorithms that split the subnodes. The inks were then classified according to these concentrations. For RF, as a number of trees are used, the 2 boundary concentrations are averaged to 3.1 wt% and 5.1 wt%. The boundary regarding F127 concentrations consists of 3 concentrations for both DT and RF. They are 8.0 wt%, 18.0 wt%, and 23.0 wt% for DT and 27.5 wt%, 7.6%, and 22.5 wt% for RF, which are different from the printability data, proving the extrapolation capability of the algorithms. For the printability map of F127/LP inks predicted by DL, the boundary of the printability window includes all 101 LP concentrations (0.0 to 10.0 wt%) demonstrating a finer granularity (Fig. 5C). The reason is that the output of the hidden layers is the possibility (0 to 1) of the input being printable, which is a continuous variable. This predicted possibility is then transformed into a binary classification (threshold as 0.5) in the output layer as the final output of the algorithm. The continuity of the predicted possibility contributes to the finer granularity of the predicted printability window. This not only proves the ability of DL to predict formulations outside the data range, but also demonstrates the higher flexibility of the DL model compared to DT and RF.

The printability maps of PCL/nHA inks predicted by DT and RF (Fig. 5D and E) are similar due to their similar working principles and a relatively small amount of data of PCL/nHA inks. The printability region predicted by RF has an extra transition at 40 w/v% PCL compared to DT, which is likely due to the diversity resulting from the number of trees in RF. The printability map predicted by DL has a lower granularity and a smoother transition, which is also observed for F127/LP inks. In addition, the printable region has extended further from the printable inks in the training data to the 0 w/v% PCL concentration region demonstrating enhanced extrapolation compared to DT and RF. This allows prediction on inks with a higher difference in concentration compared to the training data. This also results in the higher number of inks predicted as printable and is likely the reason that DL has a higher recall for predicting printability.

As mentioned previously, in terms of the prediction performance between the algorithms, RF has shown better performance than DT while there is a trade-off between higher precision (90.6%) and accuracy (88.1%) of RF and higher recall (87.3%) and better extrapolation of DL. Hence, when developing biomaterial formulations, researchers can compare the results from the printability maps generated by RF and DL. Formulations close to the central area of overlapped printable regions predicted by RF and DL have the highest likelihood to be printable. Therefore, they can be recommended for further ink development as they are likely to require less time and resources for trial-and-error process. Formulations in the unprintable regions predicted by both RF and DL are to be avoided. Formulations at the edge of the printable regions or within one printable region but outside the other are likely close to the threshold of being printable. These formulations can be favorable for certain applications. For example, hydrogel inks toward the lower limit of printability may not be suitable for printing scalable porous scaffold which requires high SF, but can be valuable for 3D printing wound dressing applications with lower requirements on SF. PCL/nHA inks with a high concentration range may have a higher chance of nozzle clogging; they can be developed to print scaffold structures that require high SF.

The printability maps generated by the ML algorithms were learned from a small number of inks to save precious time and resources for researchers. The physical meaning of different regions of the printability map was also discussed. On the other hand, the small amount of data may limit the range and accuracy of the printability map. For example, for F127/LP hydrogel inks, high concentrations of hydrogel nanocomposites (top right of printability map) could not be formulated into a homogeneous mixture due to their high viscosity. Hence, there is no upper limit for the printability map of F127/LP, but it does not necessarily mean that inks above the 40 wt% F127 and 10 wt% LP range are printable. For PCL/nHA inks, the ML algorithms have successfully predicted the upper limit as the data contain inks with concentrations too high to extrude. However, the top left region of the printability map to low PCL w/v% and high nHA remains to be explored for applications of 3D printing bone implants with high mineral content for bioactivity and stiffness. Overall, the ML algorithms have shown great potential to guide ink formulation in exploring the printability window. Data from future experiments can also be used as input to further improve the ML models forming a reinforcing circle.

## Conclusion

In this study, 210 ink formulations made from 16 biomaterials were 3D printed and their printability was assessed. The ML algorithms (DT, RF, and DL) have successfully predicted the printability of biomaterials using their formulations. All 3 algorithms have achieved >80% accuracy while RF has achieved the highest accuracy (88.1%), precision (90.6%), and F1 score (87.0%), indicating the best overall performance. DL algorithm has the highest recall (87.3%). Furthermore, the ML algorithms have successfully predicted the printability map of ink formulations. In particular, the DL algorithm provides finer granularity and enhanced extrapolation for the predicted printability window. The ML algorithms developed in this study have been shown to be a powerful tool to accelerate ink development by screening out unprintable formulations and selecting printable formulations for targeted applications. The working principles and tuning of the ML algorithms in an interdisciplinary context were elucidated to unravel the “black box” of the ML algorithms. This study sheds light on ML for researchers to realize its potential for advancements in biomaterial ink development.

## Materials and Methods

### Ink preparation for 3D printing

In this study, the data were all accumulated from wet experiments conducted in the laboratory with controlled parameters to ensure the reliability and consistency of the data used in this study. For all the polymeric inks used in this study, aqueous hydrogel inks are referred to as hydrogel-based inks, while polymer organic solvent inks are termed polymer-based inks. The materials including polymers, fillers, and solvents used to prepare the inks were either purchased from different suppliers or synthesized in the laboratory. They are listed in Table S1. The different physical and chemical properties of the solvents used are listed in Table S2. The criteria for material selection are also included in the Supplementary Materials.

The biomaterials were formulated with a range of concentrations to present the underlying pattern between ink formulation and printability for training algorithms effectively. To make hydrogel-based inks, dry hydrogel powders were mixed with de-ionized water under magnetic stirring (200 to 700 revolutions per minute [rpm]) for at least 2 h. All hydrogel-based inks were stored for at least 1 day before further tests to allow equilibration. To make polymer-based inks, polymers were dissolved in organic solvents with magnetic stirring (50 to 500 rpm) for at least 2 h. When functional fillers were used, they were mixed with solvents under magnetic stirring (700 rpm) to form a homogeneous mixture prior to the addition of hydrogels/polymers. All inks were centrifuged (2,000 rpm) for 3 min to remove air bubbles.

### 3D printing process

All 3D printing processes were operated with a DIW printer that was modified from a commercial FDM printer (Prusa i3). The original thermoplastic extruder of the FDM printer was replaced by a custom-made syringe pump extruder allowing direct extrusion of viscous inks. The nozzle diameter was 0.4 mm, the layer thickness was 0.4 mm, and the print speed was 10 mm/s. Room temperature (20 °C) was used for printing all other inks. The designed printing geometry is a 0°/90° lattice scaffold with a filament width of 0.4 mm, a fill gap of 0.8 mm, and a side length of 12 mm. Each ink was 3D printed into the 0°/90° scaffolds with 4 layers.

### Printability assessment

To assess the printability of biomaterial formulations, a total of 210 formulations with 2 ink systems (hydrogel-based and polymer-based inks) were 3D printed into 4-layer lattice scaffold structures, and their SF was evaluated. The images of the scaffolds were taken with a microscope (Leica DM 500). For hydrogel-based inks, the printed structure was assessed immediately after printing, while for polymer-based inks, the printed structure was assessed after solvent evaporation was complete. The printability of an ink formulation is determined by the SF of the 3D printed structure. To quantitatively assess the SF, this study incorporates and combines 2 important parameters used in other studies to calculate printability, the printing accuracy (PA) [60] and the printability index (*P_r_*) [40], which are calculated as follows:

$$\mathrm{PA}=\left(1-\frac{\left|A_{m}-A_{d}\right|}{A_{d}}\right)\times 100\%$$ (1)

$$P_{r}=\frac{L^{2}}{16A_{m}}$$ (2)

where *A_m_* refers to the measured pore area, *A_d_* is the designated pore area, and *L* refers to the perimeter of the pore. While PA calculates how the pore area is retained regardless of the pore shape, *P_r_* assesses how the pore shape resembles a square (designated shape), regardless of how pore area deviates from the designated value. In this study, the SF combines these 2 parameters and is calculated as their harmonic mean:

$$\mathrm{SF}=2\times \left(\frac{\mathrm{PA}\times P_{r}}{\mathrm{PA}+P_{r}}\right)\times 100\%$$ (3)

SF evaluates how the 3D printed structure maintains both area and shape of pores to determine the printability of the inks. An SF higher than 35% was considered printable. An example of the printability data matrix is shown in Table S2. The dataset consists of 20 input features, including 16 biomaterials and 4 solvents. There is one output: printability (yes or no) in the dataset.

### ML algorithms

**Fig. 6.**
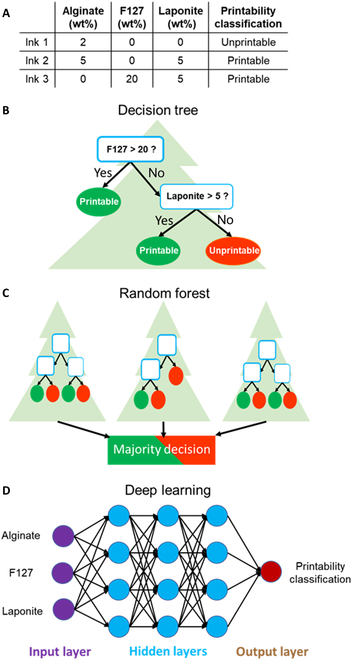
The working principles of the machine learning algorithms including DT, RF, and DL. (A) Example of a dataset of printability of hydrogel formulations. Each item (ink) is associated with several features (hydrogel type/name) and one output (printability). Schematics of the internal structures of machine learning algorithms include (B) DT, (C) RF, and (D) DL after learning from the dataset.

In this study, DT, RF, and DL algorithms were developed in Python v3.8.8 to predict the printability of an ink. RF was developed using the Scikit-learn package and DL was developed using the Keras package. The working principle of the algorithms is shown in Fig. 6. The dataset is split into training dataset *X*, and testing dataset (holdout set) *Y* in a ratio of 7:3. The algorithms were trained using the training dataset and subsequently tested on the testing dataset, unseen to the model, to assess their generalization ability [58,59]. During testing, the printability of the biomaterial formulations in the testing dataset was predicted using the trained ML models and the prediction results were assessed. The complexity of ML models is an important aspect influencing the learning capability and, in turn, the generalization ability of the algorithms [61]. Hence, the key hyperparameters of the algorithms were tuned to generate the algorithms with varying degrees of complexity, and their effect on the prediction performance of the ML models was examined. The optimal configurations of ML models were then determined through the investigation of the complex interplay between algorithmic complexity and model performance.

### Decision tree

The DT algorithm has played a major role in data classification, due to its simplicity and effectiveness in aggregating diverse types of data to make accurate predictions [62,63]. A DT contains nodes and branches that lead to other nodes. An item is classified by a DT by following the path from the top node (root) to one of the bottom nodes (leaf) where its classification is assigned. There is a feature-related question in each node to determine which branch the item should follow. The Gini index is used to measure the impurity of nodes.

$$\text{Gini}=1-\sum_{i=1}^{c}P_{i}^{2}$$ (4)

where *c* is the number of classes (2 in this study) and *P_i_* are the fraction of the items in the classes. The effect of the MNF to consider for splitting the branches and the MNL in the tree on the performance of DT was studied.

### Random forest

RF consists of an ensemble of DTs each trained with data resampled from the training data X with the bootstrap method: samples are randomly chosen to reach the same size with replacement (i.e., the same sample can be chosen multiple times). On average, 63.2% of training data are chosen to train while the other 36.8% are omitted [64]. Such randomization enhances the diversity of DTs and reduces the chance of overfitting [62]. For predicting printability, the final prediction was determined by a majority vote from the DTs. Each tree was trained by data randomly resampled from the training data of 147 formulations (70% of total data) with replacements. The effect of the MNL in each DT and the number of DTs in the RF algorithm on its performance was studied.

### Deep learning

DL is designed using numerous layers of ANNs, each of which provides a different interpretation of the data that has been fed to them [65,66]. ANN is an ML algorithm inspired by biological neural networks. It consists of interconnected computational units named artificial neurons that imitate the biological neurons of the human brain [67]. ANNs consist of one input layer, multiple hidden layers, and one output layer. The input layer receives input data (biomaterial formulations or printability profiles) and transfers a processed value to the next hidden layer. The hidden layers are considered as the computational engine. Neurons in the hidden layer receive input values from other neurons in the previous layer, combine them with weights, summate them together with a bias before applying an activation function, and lastly outputs the resultant value [65]. The output layer is the last layer that transforms the information received from the hidden layer into outputs [68].

For predicting printability, the input layer consists of 20 nodes, each assigned to an input feature. There is one node in the output layer for outputting printability (yes/no). The batch size is the number of items passing through the model in one batch during training, which was set as 16. Epoch is the process of passing a batch of items through the neural networks and running a backpropagation (calculating an error and updating the weights accordingly). The epoch size was set as 500. The batch size and epoch number were set manually from preliminary tests. Higher batch size increased testing speed but reduced accuracy. The epoch number was set as a sufficient number for the neural networks to reach convergence, that is, any further increase did not result in higher performance but reduced testing speed. The algorithm was tested with a range of node numbers in hidden layers and a range of hidden layer numbers in order to research their influence and optimize the algorithm.

### Evaluation metrics

**Fig. 7.**
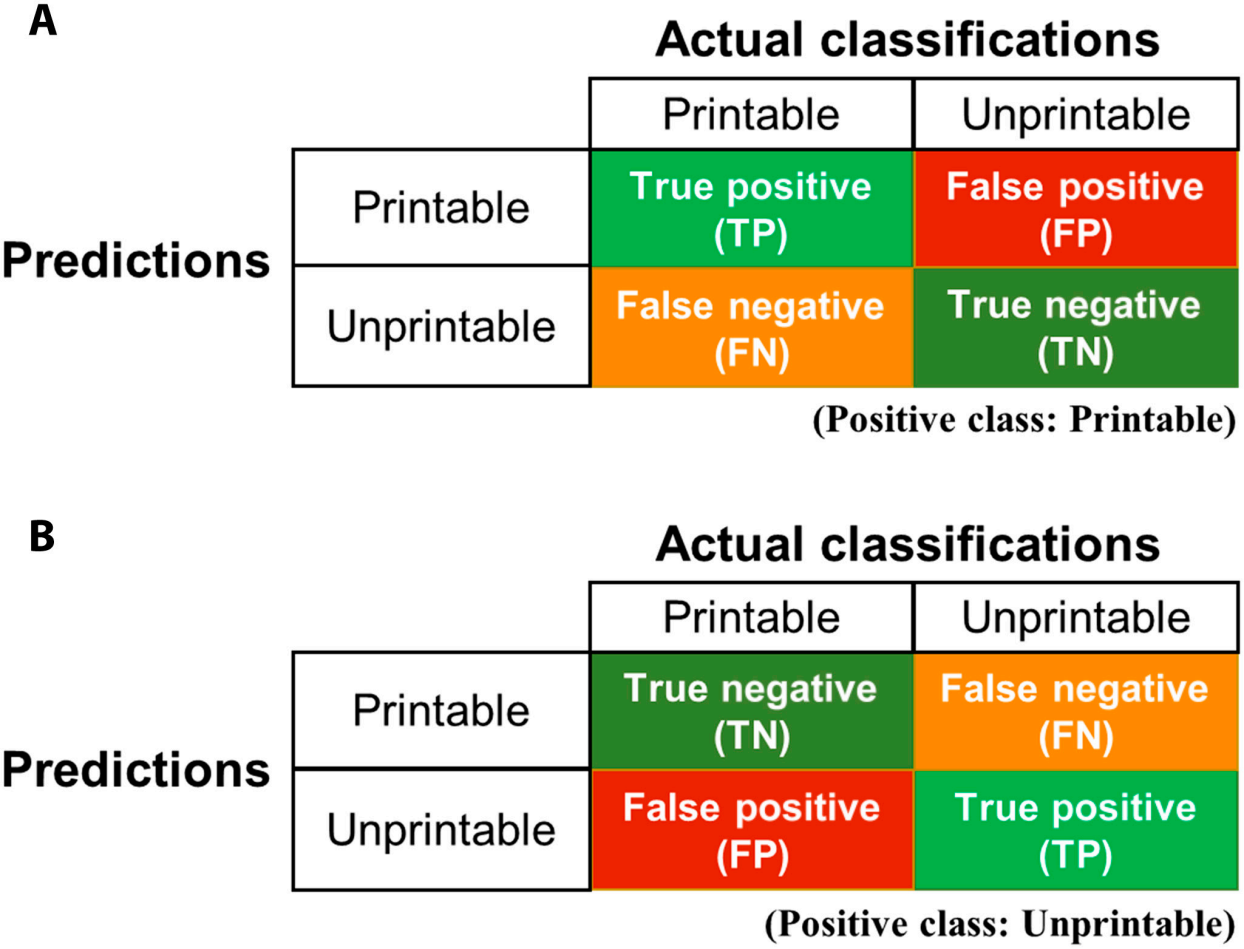
The confusion matrix for ink printability classification. Each row of the matrix represents predicted instances of each class, while each column represents the actual instances of each class. (A) The confusion matrix with the printable class as the positive class and (B) the confusion matrix with the unprintable class as the positive class.

After training the ML algorithms with the training dataset, they were tested with an unseen testing dataset to assess their generalization ability. The prediction results were summarized in a confusion matrix as compared to the actual classifications (Fig. 7). There are 2 output classes, printable and unprintable. For assessing the prediction on printable inks, the printable class is considered as the positive class in the confusion matrix (Fig. 7A), and for assessing the prediction on unprintable inks, the unprintable class is considered as the positive class (Fig. 7B).

The 4 values in the confusion matrix were then used to calculate evaluation metrics including accuracy, precision, recall, and F1 score to assess the prediction performance of the ML algorithms [69]. Accuracy is calculated as the percentage of correctly predicted cases among all predictions. Precision is the ratio between correctly predicted positive cases among all cases predicted as positive. Recall refers to the percentage of correctly predicted positive cases among all positive cases. F1 score is a weighted average between precision and recall, which provides a balanced measure of the performance of the ML algorithms in predicting positive cases. These parameters are calculated as below:

$$\text{accuracy}=\frac{\mathrm{TP}+\mathrm{TN}}{\mathrm{TP}+\mathrm{TN}+\mathrm{FP}+\mathrm{FN}}$$ (5)

$$\text{precision}=\frac{\mathrm{TP}}{\mathrm{TP}+\mathrm{FP}}$$ (6)

$$\text{recall}=\frac{\mathrm{TP}}{\mathrm{TP}+\mathrm{FN}}$$ (7)

$$F1=2\times \frac{\text{precision}\times \text{recall}}{\text{precision}+\text{recall}}$$ (8)

where TP are true positives, FP are false positives, and FN are false negatives.

Precision, recall, and F1 score focus on evaluating the performance of the algorithms in predicting positive cases as they are all proportional to true positives. Hence, in this study, they were calculated separately with the 2 confusion matrices. To assess prediction performance on printable inks, the printable class is considered the positive case and evaluation metrics were calculated using (Fig. 7A). Similarly, the unprintable class is considered the positive case (Fig. 7B) to calculate the evaluation metrics for assessing the prediction performance on unprintable inks. This allows for a more comprehensive analysis on the prediction performance to facilitate the application of these algorithms in various contexts, thereby broadening their utilization.

### Printability window prediction

The printability window of F127/LP hydrogel nanocomposites and PCL/nHA polymer nanocomposites in DCM was predicted using ML algorithms (DT, RF, and DL) simulating the practical use of the ML algorithms for ink development. The ML algorithms were trained from all 210 formulations in the printability data. A total of 45,511 testing formulations of F127/LP hydrogel nanocomposites (F127: 0.0 to 40.0 wt% and LP: 0.0 to 10.0 wt%) were input into the ML algorithms to predict the printability map of F127/LP inks. For PCL/nHA inks, 350,001 testing formulations (PCL: 0 to 70 w/v% and nHA: 0 to 60 w/v%) were used.

## Data Availability

All data needed to evaluate the conclusions of the study are available.

## Supplementary Materials

Supplementary 1 Figs. S1 and S2 Tables S1 to S3 References [70-72]
