# Supplementary material for: Machine Learning in Predicting Printable Biomaterial Formulations for Direct Ink Writing
Source: Research (Wash D C). 2023 Jul 18;6:0197. doi: 10.34133/research.0197 (PMC10353544; doi:10.34133/research.0197)
Supplement: Supplementary 1 — Figs. S1 and S2 Tables S1 to S3 References [70-72] [file research.0197.f1.docx]

Supplementary materials

Machine Learning in Predicting Printable Biomaterial Formulations for Direct Ink Writing

Hongyi Chen^1,2^, Yuanchang Liu^1^, Stavroula Balabani^1,3^, Ryuji Hirayama^2^, and Jie Huang^1*^

^1^Department of Mechanical Engineering, University College London, London, UK.

^2^Department of Computer Science, University College London, London, UK.

^3^Wellcome-EPSRC Centre for Interventional Surgical Sciences (WEISS), University College London, London, UK

^*^Address correspondence to: [jie.huang@ucl.ac.uk](mailto:jie.huang@ucl.ac.uk)

**Results**

Part of the decision tree generated by DT algorithm is shown in Figure S1. An item follows the path from the top node (root) to a bottom node (leaf) where it is classified. The feature-related question (yes/no) in each non-leaf node guides the partition of the items to subnodes, where the sum of the Gini index decreases. The Gini index in the nodes at the bottom (leaves) is zero, meaning the items in the leaves all belong to one classification.


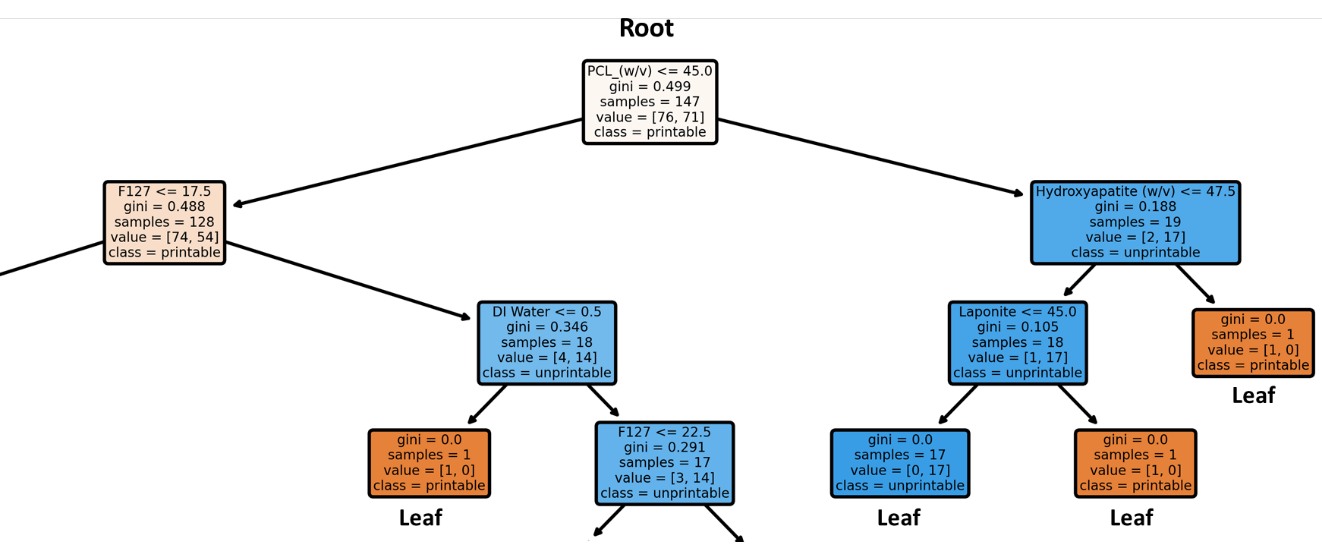


Figure S1. Part of the decision tree generated by the DT algorithm from training with the training dataset of 147 formulations (70% of total data) for predicting printability. The top line is the question for partitioning items in the decision tree. Items with the answer “yes” follow the path to the left subnode while the item with the answer “no” go to the right subnode. Gini is the Gini index measuring the impurity of the node and “Class” is the classification of the items in the node. Gini starts at 0.499 at the root and decreased to 0 at the bottom nodes (leaves) where items belong to only one class. ‘Samples’ refers to the number of samples that went through the node during training and the two numbers in the “value” refers to the number of printable (left) and unprintable (right) items in the node according to the prediction result.

The biomaterials used to prepare hydrogel-based inks and polymer-based inks were either purchased from different suppliers or synthesized in the lab as shown in Table S1.

Each biomaterial has been formulated with a range of concentrations to present the underlying pattern between ink formulation and printability for training algorithms effectively.

**Table S1:** List of materials for ink preparation.

| Materials | Producers |
| --- | --- |
| Poly(ethylene oxide) (Mw: 60k) | Sigma Aldrich, UK |
| Polyvinylpyrrolidone (Mw: 10k) | Sigma Aldrich, UK |
| Poly(ethylene oxide) (Mw: 20k) | Sigma Aldrich, UK |
| (Hydroxypropyl)methyl cellulose | Sigma Aldrich, UK |
| Polycaprolactone (Mw:80k) | Sigma Aldrich, UK |
| Xanthan gum from Xanthomonas campestris | Sigma Aldrich, UK |
| Polyvinylpyrrolidone_40K (Mw: 40k) | Sigma Aldrich, UK |
| Pluronic F127 (Mw: 12k) | Sigma Aldrich, UK |
| Gelatin | Sigma Aldrich, UK |
| Alginic acid sodium salt from brown algae (medium viscosity) | Sigma Aldrich, UK |
| Poly(vinyl alcohol) (Mw 89000-98000, 99+% hydrolyzed) | Sigma Aldrich, UK |
| Laponite RD | BYK Additives & Instruments |
| Hydroxyapatite microparticles | Synthesized in lab |
| Hydroxyapatite nanoparticles (≥96%, 60 nm±10 nm) | Sigma Aldrich, UK |
| Nanoclay, hydrophilic bentonite (≤25 μm) | Sigma Aldrich, UK |
| Metal organic framework nanoparticles (MOF) | Synthesized in lab |
| Dichloromethane (anhydrous, ≥99.8%) | Sigma Aldrich, UK |
| Ethanol absolute | VWR |
| Chloroform (anhydrous, ≥99%) | Sigma Aldrich, UK |

A range of bioactive inorganic fillers, such as hydroxyapatite and laponite, were used in the study. The size varies from nano to micrometres and the morphology includes discs, spheres, rods and cubes, as shown from the SEM micrographs in Fig S2.


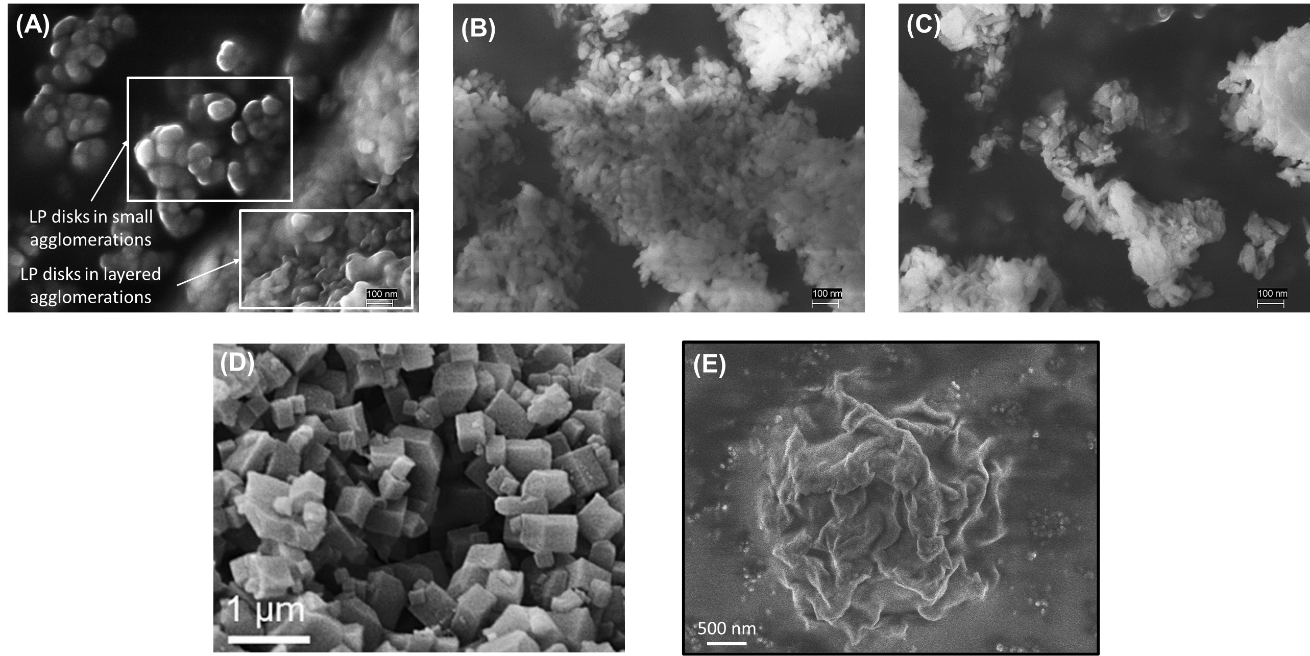


Figure S2 SEM images of the fillers used in this study including (a) Laponite, (b) hydroxyapatite microparticles, (c) hydroxyapatite nanoparticles, (d) metal-organic framework, and (e) a bentonite nanoclay cluster. (d) is adapted from Alotaibi et al. [3] with permission.

Different volatile organic solvents including dichloromethane (DCM), ethanol and chloroform were used. They also have a range of volatilities which impacts the rate of solvent evaporation and thus the solidification and shape fidelity of the printed structure. The different physical and chemical properties of the solvents used are listed in Table S2.

Table S2 Different physical and chemical properties of the solvents

| Properties | DCM | Ethanol | Chloroform | DI water |
| --- | --- | --- | --- | --- |
| Boiling Point (°C) | 39.6 | 78.3 | 62.1 | 100 |
| Density (kg/m^3^) | 1326 | 785 | 1480 | 997 |
| Vapor Pressure (kPa) | 58.40 | 5.90 | 20.90 | 2.4 |
| Dipole moment (D) | 1.14 | 1.66 | 1.15 | 1.84 |
| Dielectric constant | 8.93 | 24.55 | 4.81 | 80.1 |
| Viscosity (mPa s) | 0.41 | 1.074 | 0.563 | 0.89 |
| Surface tension(mN/m) | 27.36 | 21.97 | 26.56 | 72.7 |
| Relative evaporation rate  (Butyl acetate = 1) | 14.5 | 1.7 | 8.8 | 0.36 |

For assessing the printability of biomaterial formulations, 210 formulations with 2 ink systems were 3D printed and their printability was assessed. Example of the printability data matrix is shown in Table.S2. In the table, the values for each ink formulation are the concentrations (wt% or w/v%) of each biomaterial; while the values for solvents are either 0 or 1, indicating whether the solvent is used. The dataset is split into training data *X*, and testing data *Y* in a ratio of 7:3.

**Table.S3** Example of the data matrix for predicting printability classifications with ink formulations.

|  | | Training data (70%) | | | Testing data (30%) | |
| --- | --- | --- | --- | --- | --- | --- |
|  |  | Ink 1 | Ink 2 | … | | Ink 210 |
| Input | PEO_60K (w/v) | 0 | 0 | … | | 0 |
|  | PVP_10K (w/v) | 0 | 0 | … | | 0 |
|  | PEO_20K (w/v) | 0 | 0 | … | | 0 |
|  | MC (w/v) | 0 | 0 | … | | 0 |
|  | PCL (w/v) | 15 | 0 | … | | 50 |
|  | Xanthan Gum (w/v) | 0 | 0 | … | | 0 |
|  | PVP_40K (w/v) | 0 | 0 | … | | 0 |
|  | F127 (wt%) | 0 | 20 | … | | 0 |
|  | Gelatin (wt%) | 0 | 0 | … | | 0 |
|  | Alginate (wt%) | 0 | 0 | … | | 0 |
|  | PVA (wt%) | 0 | 0 | … | | 0 |
|  | Laponite (wt%) | 0 | 4 | … | | 0 |
|  | MicroHA (wt%) | 0 | 0 | … | | 0 |
|  | NanoHA (wt%) | 0 | 0 | … | | 20 |
|  | Bentonite (wt%) | 0 | 0 | … | | 0 |
|  | MOF (wt%) | 0 | 0 | … | | 0 |
|  | DCM | 1 | 0 | … | | 0 |
|  | Ethanol | 0 | 0 | … | | 0 |
|  | Chloroform | 0 | 0 | … | | 1 |
|  | DI Water | 0 | 1 | … | | 0 |
|  | Temperature (°C) | 20 | 37 | … | | 20 |
| Output | Printability classification | no | yes | … | | yes |

**References**

[1] Kroon, M., W.L. Vos, and G.H. Wegdam, Structure and formation of a gel of colloidal disks. *Physical Review E*, 1998. **57**(2): p. 1962-1970. 10.1103/PhysRevE.57.1962.

[2] Jatav, S. and Y.M. Joshi, Chemical stability of Laponite in aqueous media. *Applied Clay Science*, 2014. **97-98**: p. 72-77. <https://doi.org/10.1016/j.clay.2014.06.004>.

[3] Alotaibi, H., E. Chung, S.H. Chung, G. Ren, V. Singh, and J. Huang, Sustainable γ-cyclodextrin frameworks containing ultra-fine silver nanoparticles with enhanced antimicrobial efficacy. *Carbohydrate Polymers*, 2023. **304**: p. 120516. <https://doi.org/10.1016/j.carbpol.2022.120516>.
